# Reducing Falls Among Community-Dwelling Older Adults From Clinicians’ Perspectives: A Systems Modeling Approach

**DOI:** 10.1093/geroni/igad077

**Published:** 2023-07-20

**Authors:** Vanessa Jean Wen Koh, David B Matchar, Angelique Wei-Ming Chan, June May-Ling Lee, Wei Xuan Lai, Dulcie Rosario, Anne George, Vanda Ho, Noor Hafizah Bte Ismail, Christopher Tsung Chien Lien, Reshma A Merchant, Shuyan Melissa Tan, Chek Hooi Wong, Tianma Xu

**Affiliations:** Programme in Health Services and Systems Research (HSSR), Duke-NUS Medical School, Singapore, Singapore; Centre for Ageing Research and Education (CARE), Duke-NUS Medical School, Singapore, Singapore; Programme in Health Services and Systems Research (HSSR), Duke-NUS Medical School, Singapore, Singapore; Department of Medicine (General Internal Medicine), Duke University Medical Center, Durham, North Carolina, USA; Programme in Health Services and Systems Research (HSSR), Duke-NUS Medical School, Singapore, Singapore; Centre for Ageing Research and Education (CARE), Duke-NUS Medical School, Singapore, Singapore; Centre for Ageing Research and Education (CARE), Duke-NUS Medical School, Singapore, Singapore; Programme in Health Services and Systems Research (HSSR), Duke-NUS Medical School, Singapore, Singapore; Centre for Ageing Research and Education (CARE), Duke-NUS Medical School, Singapore, Singapore; Rehabilitation Services, Changi General Hospital, Singapore, Singapore; Department of Geriatric Medicine, National University Hospital, Singapore, Singapore; Geriatric Medicine, Tan Tock Seng Hospital, Singapore, Singapore; Department of Geriatric Medicine, Changi General Hospital, Singapore, Singapore; Division of Geriatric Medicine, Department of Medicine, National University Hospital, Singapore, Singapore; Rehabilitation Services, Changi General Hospital, Singapore, Singapore; Programme in Health Services and Systems Research (HSSR), Duke-NUS Medical School, Singapore, Singapore; Health and Social Sciences Cluster, Singapore Institute of Technology, Singapore, Singapore

**Keywords:** Accidental falls, Fall prevention, Group model building, Implementation, Systems dynamics

## Abstract

**Background and Objectives:**

Falls among older adults are a significant health problem globally. Studies of multicomponent fall prevention programs in randomized controlled trials demonstrate effectiveness in reducing falls; however, the translation of research into the community remains challenging. Although there is an increasing interest to understand the factors contributing to implementation barriers, the dynamic relationships between factors are less well examined. Furthermore, evidence on implementation barriers from Asia is lacking as most of these studies originate from the West. As such, this study aims to engage stakeholders in uncovering the factors that facilitate or inhibit implementing community-based fall prevention programs in Singapore, with a focus on the interrelationship between those factors.

**Research Design and Methods:**

Health care professionals familiar with fall prevention programs were invited to discuss the enablers and challenges to the implementation. This effort was facilitated using a systems modeling methodology of Group Model Building (GMB) to share ideas and create a common conceptual model of the challenges. The GMB employs various engagement techniques to draw on the experiences and perceptions of all stakeholders involved.

**Results:**

This process led to the development of a Causal Loop Diagram (CLD), a qualitative conceptual model of the dynamic relationships between the barriers and facilitators of implementing fall prevention programs. Results from the CLD show that implementation is influenced by two main drivers: health care provider factors that influenced referrals, and patient factors that influenced referral acceptance and long-term adherence. Key leverage points for potential interventions were identified as well.

**Discussion and Implications:**

The overall recommendation emphasized closer coordination and collaboration across providers to ensure sustainable and effective community-based fall prevention programs. This has to be supported by a national effort, involving a multidisciplinary stakeholder advisory group. These findings generated would be promising to guide future approaches to fall prevention.

**Translational Significance:** Translating fall prevention research into the community has been challenging due to unique implementation barriers. Although there is interest in studying the implementation aspects of programs, the dynamic relationships are less understood. Through a systems dynamics methodology of group model building, health care professionals were engaged in a participatory approach to building a shared understanding of the fall prevention system in Singapore. A Causal Loop Diagram was developed to visualize the complexities involved. By understanding the dynamic relationships, potential policy targets were identified and recommendations for the development of a comprehensive national strategy, encapsulating the facets of fall prevention, were proposed.

Falls among community-dwelling older adults are a significant health concern globally. About one in four older adults above the age of 65 fall annually, with 10% of fallers suffering from recurrent and injurious falls respectively (Salari et al., 2022; Vaishya & Vaish, 2020). Falls are associated with many adverse consequences, including fear of falling, functional decline, reduced quality of life, and prolonged risk of hospitalization (Salari et al., 2022; Salvà et al., 2004).

Studies have shown that multicomponent fall prevention programs are a comprehensive and effective way to prevent falls in older adults (Dautzenberg et al., 2021; Tricco et al., 2017). A recent meta-analysis of 192 randomized controlled trials demonstrated that multifactorial interventions were associated with a 13% reduction in falls rate compared to the usual care (Dautzenberg et al., 2021). Exercise-based fall prevention programs such as Otago (Albornos-Muñoz et al., 2018) and Stepping On (Sherrington et al., 2016) have demonstrated effectiveness in reducing falls. In Singapore, previous exercise-based interventions such as SAFE have also demonstrated effectiveness in reducing injurious falls (Matchar et al., 2017).

Although the evidence from research supports the effectiveness and efficacy of fall prevention programs, implementing and sustaining these initiatives in the community are challenging (Horne et al., 2014; Yardley et al., 2006). A key challenge is that activities central to the programs are carried out in multiple settings (e.g., in the community or outpatient medical care), and by multiple providers (e.g., doctors, physiotherapists, and trainers in program centers) in an uncoordinated and fragmented manner (Ganz et al., 2008). As such, the quantity and quality of fall prevention programs, coordination among health care providers, and patient factors influence the success of interventions (Dykeman et al., 2018; Child et al., 2012).

In Singapore, the development of a robust, multifaceted fall prevention program in the community is still largely in its infancy. Most fall prevention programs, which include a clinical model of risk assessments and referrals done by clinicians to physiotherapists or occupational therapists, are currently conducted in geriatric clinics (Shyamala et al., 2015). Due to the rapidly aging population, there is increasing attention being paid to implementing fall prevention beyond the hospital and into primary care and the community (He & Tang, 2021). Currently, most multicomponent fall prevention programs in the community are research-based programs. These include group-based programs such as SAFE (Matchar et al., 2017) and STEADY-FEET (Ong et al., 2022), where patients are recruited from hospitals or the community. Additionally, single-component fall prevention initiatives are available and conducted through organizations such as the Health Promotion Board or Agency for Integrated Care, which includes community outreach, exercise programs, or home safety assessments (Agent for Integrated Care, n.d.; HealthHubSG, n.d.).

It seems clear that the potential impact of fall prevention programs is constrained by implementation barriers. Although these implementation barriers have been increasingly studied (Goodwin et al., 2011; McMahon et al., 2022; Reckrey et al., 2021), the dynamic relationships between factors have not been explored. Some examples of dynamic relationships between implementation barriers include resource constraints affecting program effectiveness and poor communication between stakeholders impeding the execution of programs (Tinetti et al., 2006). Understanding the dynamics between factors would provide insight into designing specific solutions to overcome challenges as addressing one implementation barrier may require addressing others as well. The systems dynamics approach has been increasingly recognized as a powerful method to understand complex health issues (Homer & Hirsch, 2006). This approach is suitable to visualize the relationships and feedback mechanisms and identify key leverage points for interventions (Homer & Hirsch, 2006). In addition, implementation barriers may be unique to different regions and countries. Currently, studies on the implementation barriers are mostly from the West (Child et al., 2012; van Rhyn & Barwick, 2019), making it vital to investigate the challenges in translating research into practice in an Asian context. As Singapore is a multiethnic country with a developed health care system, findings from this study will provide a unique and comprehensive insight into the implementation challenges in an Asian setting. These would be useful to inform the development and implementation of future fall prevention programs.

This research aims to engage key stakeholders familiar with fall prevention through a system dynamics modeling methodology, Group Model Building (GMB). GMB is a participatory approach used to engage stakeholders, reach a consensus, and build a shared understanding of the system (Siokou et al., 2014). As health care professionals (HCPs) oversee patient care, they would be familiar with issues regarding the effectiveness of programs and feasibility issues during implementation. Hence, we aim to engage HCPs to examine the nature of implementing fall prevention programs in Singapore and highlight important factors and relationships within the systems. Finally, a shared mental model of the barriers and facilitators of implementing fall prevention programs will be generated to visualize the complexities and feedback perspectives to inform future interventions. This study is the first part of a series of efforts to engage various stakeholders involved in fall prevention to develop recommendations for a comprehensive fall prevention strategy.

## Method

Group Model Building has been increasingly used in health systems research to understand the complexities of policy initiatives, community-based programs, and mechanisms of primary care in health and chronic disease prevention (Ansah et al., 2018; Gerritsen et al., 2020). The complex nature of the problem requires the development of shared mental models to gain a whole-system perspective. GMB is a participatory form of systems dynamics modeling that engages stakeholders and facilitates understanding of relationships that determine system behaviors (Király & Miskolczi, 2019). Through formal exercises during the GMB, the dynamics of implementing fall prevention programs were explored, and a conceptual model was created after the session. The GMB utilizes activities from ScriptMap (Ackermann et al., 2011), which are formal exercises carried out to engage stakeholders to elicit variables, hypotheses, and the structure of conceptual models. Suitable scripts were selected based on the objectives of the GMB session.

### Outcome

The outcome of the GMB was to develop a qualitative model elucidating the facilitators and barriers in implementing fall prevention programs. The model aims to describe older adults’ engagement in community-based fall prevention programs, which include outpatient clinics, clinics in primary care settings, and community-based institutions such as senior care centers.

### Setting

The research team conducted a half-day workshop at Duke-NUS Medical School, Singapore on November 2022. Sixteen clinician-scientists, clinicians, researchers, and allied health professionals attended the GMB session. They represented the following institutions: National University Hospital, Tan Tock Seng Hospital, SingHealth Community Hospital, Changi General Hospital, Singapore Institute of Technology, and the Geriatric Education and Research Institute. These stakeholders included six medical doctors, two physiotherapists, one occupational therapist, and seven researchers. All stakeholders consented to audio recordings and photography during the session.

### Design

Group activities were conducted with stakeholders during the workshop. Exercises were planned based on suitable scripts from ScriptMap and were facilitated by experienced research team members (Ansah et al., 2023; Matchar et al., 2016). The activities were designed to promote participation and discussion from stakeholders. This process involved stakeholders building on each other’s ideas to enhance their shared understanding of the factors influencing the implementation of fall prevention programs in Singapore. The half-day GMB was divided into two sessions. The first session focused on variable elicitation, where stakeholders were asked to list the variables that facilitated or impeded the effective implementation of fall prevention programs. In the second session, stakeholders were asked about their policy recommendations and other plausible recommendations that could address the barriers identified. During these exercises, facilitators also explored and clarified definitions and the interdependencies among the factors. The GMB exercises conducted are summarized in Supplementary Table 1.

### GMB Exercises

Three exercises formed the basis of the workshop. After introducing the agenda and icebreaker activities, the stakeholders were engaged in the following activities: outcome elicitation, variable elicitation, and exploring policy options.

#### Exercise 1: Outcome elicitation

Stakeholders were asked to elicit key outcomes of fall prevention programs they were interested in to assess the effectiveness of programs. Stakeholders were presented with the question: “What are the key outcomes are you interested in when addressing falls?” In a round-robin fashion, stakeholders shared one outcome at a time and the process was repeated for all individuals. The facilitators of the workshop clarified definitions and identified how they could be measured. At the end of the activity, stakeholders were asked to prioritize key outcomes of interest (Supplementary Figure 1).

#### Exercise 2: Variable elicitation

The objective of this exercise was to elicit causal factors influencing implementation issues surrounding fall prevention programs. A guiding question was used to facilitate the discussion: “Based on your own experiences, what are the factors that promote or hinder the implementation of fall prevention programs?” Stakeholders were asked to list all the facilitators and barriers influencing the effective implementation of fall prevention programs based on their personal experiences. Stakeholders were given post-it notes to list down the variables, with one variable per post-it note. After which, in a round-robin fashion, individuals were allowed to present one factor they indicated. Facilitators probed to clarify the definition and causal relationships of variables. This process of probing and clarification is an important aspect of GMB as it ensures that the model developed accurately reflects stakeholders’ understanding of the system. Stakeholders engage in an open dialogue to identify areas of agreements or disagreements, uncover potential biases and underlying assumptions. The process of probing is guided by the Categories of Legitimate Reservation (CLR) framework to ensure variable existence and causal relationships described are well-defined (Dettmer, 1997). A variable is clearly described when a coherent story and hypothesis can be made of the variable, and its cause and effect on effective implementation can be explicated. This process was repeated for all stakeholders and variables presented. Only after thorough clarification and agreement with all stakeholders and research team members in the group would the factor be listed on a post-it note and affixed onto a wall. The research team then clustered the variables into eight groups: patient attitude, patient knowledge, patient perceptions, accessibility of program, availability of the program, characteristics of the program, community building, and family and societal norms (Supplementary Figure 2).

#### Exercise 3: Exploring policy options

The objective of this group exercise was to identify leverage points for intervention and discuss possible policy recommendations. Stakeholders were asked a guiding question: “What are some recommendations that can help increase the success of fall prevention programs?” Stakeholders discussed recommendations as a group and were asked to clarify how that recommendation affected the facilitators and barriers previously identified, and how that related to older adults’ participation and engagement in fall prevention programs. Similarly, the process of probing and clarification was used with each variable brought up by stakeholders. They were also asked to provide examples or anecdotes of the recommendations suggested. Once the variable has been clarified, the facilitator wrote the variable on a post-it note and affixed it to the wall to indicate that consensus has been achieved. This process was repeated for all variables discussed during this exercise. The policy recommendations identified are presented in Supplementary Figure 3.

### Causal Loop Diagram

A qualitative Causal Loop Diagram (CLD) was developed by the research team using the variables elicited from the GMB. The CLD was developed using Vensim (Ventana Systems, Inc.). An overview of the variables is presented in Supplementary Figure 4. A CLD was selected to represent feedback loops and interpret underlying dynamics between variables and their effect on older adults’ engagement in fall prevention programs.

To develop the CLD, variables and causal relationships were further evaluated using the set of rules outlined in the CLR (Dettmer, 1997). Applying the CLR to the entities described ensures that the assumptions, the existence of the entity, and causal relationships are accurate and well-defined. Multiple perspectives, potential biases, all other relevant entities and causal relationships in the systems, and the possibility of additional causes and effects would be considered. This thorough process ensures that as the entities in the CLD, the causal relationships, connections, and feedback loops were clearly defined. The preliminary model was also shared with stakeholders to ensure variables and relationships elucidated were consistent with their shared understanding.

Essential to systems dynamics is the idea that systems behavior emerges as reinforcing and balancing feedback loops that propagate or counterbalance the feedback system (Richardson, 2011; Sterman, 2002). A reinforcing loop is described when the effect of a change amplifies other variables within the loop, where the increase of a variable leads to a further increase in itself. On the other hand, a balancing loop is described when the increase of a variable counteracts itself, leading to a decrease in the variable. The feedback loops are denoted as “R” and “B” for reinforcing and balancing loops, respectively, and are numbered in the CLD for ease of reference (i.e., Reinforcing Loop 1: R1, Balancing Loop 1: B1). The changes in the variables are described by polarities on arrows, where a positive sign “+” indicates a direct relationship and a negative sign “−” indicates an inverse relationship. A positive “+” causal link shows that two variables change in the same direction, whereas a negative “−” polarity indicates opposing relationships where a change in one variable results in the opposite change in the other (i.e., an increase in one variable results in a decrease in the other variable and vice versa; Cavana & Mares, 2004; Lin et al., 2020). Symbols found in a typical CLD are summarized in Supplementary Table 2.

### Substantiating Quotations and Examples

To ensure objectivity in presenting these qualitative findings, the quotations and examples were selected based on relevance to the research, applicability to Singapore’s fall prevention setting, and frequency of occurrence. Additionally, stakeholders were also consulted on the CLD and accompanying explanations to ensure that they were reflective of their experiences.

## Results

**Figure 1.**
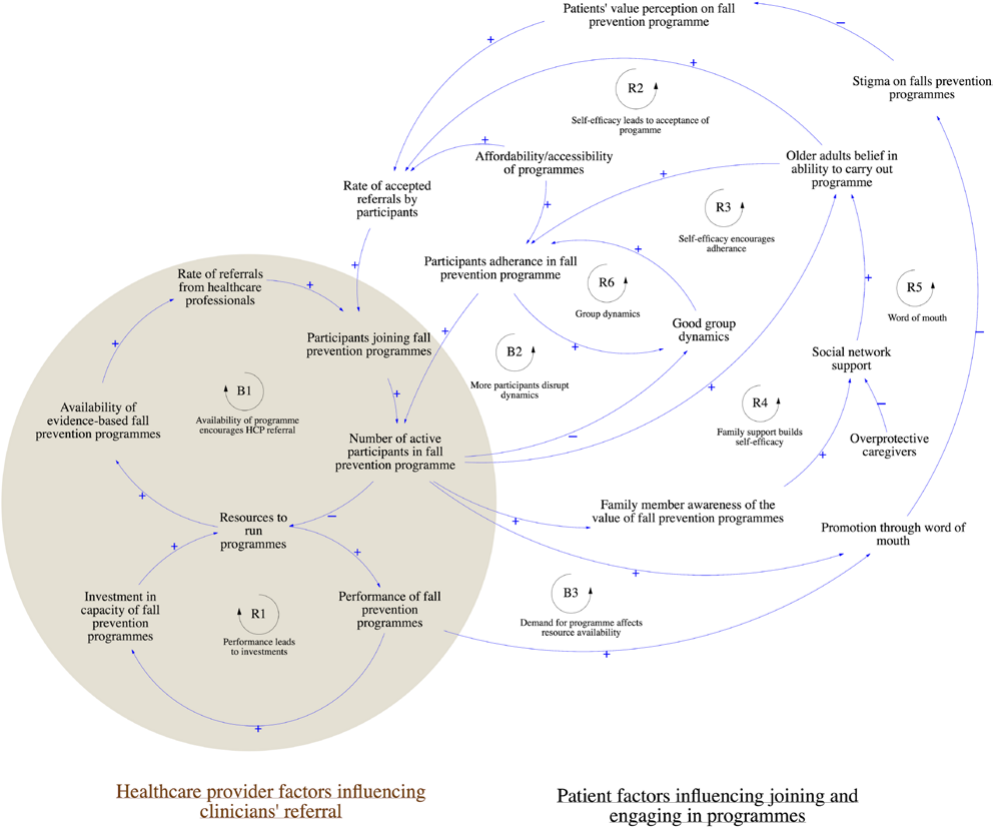
Full Causal Loop Diagram illustrating dynamic relationships between facilitators and barriers influencing the implementation of community-based fall prevention program. B1: Balancing Loop 1, R1: Reinforcing Loop 1, B2: Balancing Loop 2, R2: Reinforcing Loop 2, B3: Balancing Loop 3, R3: Reinforcing Loop 3, R4: Reinforcing Loop 4, R5: Reinforcing Loop 5, R6: Reinforcing Loop 6. Explanations of the key feedback loops are provided in Table 1.

This section describes the conceptual model developed with stakeholders. The conceptual model is divided into two sectors: (1) health care provider factors influencing clinicians’ referrals and (2) patient factors influencing joining and engaging in programs from clinicians’ perspectives. Figure 1 illustrates the full CLD, and Table 1 summarizes the key feedback loops.

**Table 1.** Identified Feedback Loops From the Concept Model

Feedback loops	Description
B1: Availability of program encourages health care professional (HCP) referral	With greater resources to run programs, the availability of fall prevention programs would increase. This would promote the rate of referrals from HCPs leading to an overall increase in the number of participants in the program. However, resources may be limited with more participants in the program.
R1: Performance leads to investments	Greater performance of fall prevention programs encourages investment in the capacity for fall prevention programs, hence increasing resource availability to run programs. With more resources, performance of program will see an increase as well.
R2: Self-efficacy leads to acceptance of program	Older adults’ initial self-belief in carrying out fall prevention programs encourages participants to accept referrals to join programs.
R3: Self-efficacy encourages adherence	Older adults’ self-belief in carrying out fall prevention programs promotes adhering to programs. Being engaged and carrying out programs reinforce their self-beliefs.
R4: Family support builds self-efficacy for programs	Family awareness of the importance of fall prevention can increase support for fall prevention programs. This increases social support for older adults through emotional, functional, and financial support. This can encourage participants to accept referrals and to adhere to program regimens.
B3: Demand for program affects resource availability	High performance of fall prevention programs will be promoted by word of mouth within the community. This can increase the value perception of fall prevention programs, hence, increasing participant acceptance rates. However, with more participants, this may place a constraint on available resources, which may reduce the performance of programs.
R5: Word of mouth	Greater active participation in programs directly affects the promotion of programs through word of mouth. This can increase older adults’ value perception by decreasing the community stigma of fall prevention programs. Greater value perception increases the rate of accepted referrals from older adults, which can ultimately increase participants joining and engaging in fall prevention programs.
R6: Group dynamics	Good group dynamics encourage participants to adhere in fall prevention program. Adherence over time also maintains and promotes good group dynamics.
B2: More participants disrupt dynamics	Good group dynamics encourage adherence to programs and increase the number of participants in programs. However, with more people joining programs, this may disrupt group dynamics.

### Health Care Provider Factors Influencing Clinicians’ Referrals

**Figure 2.**
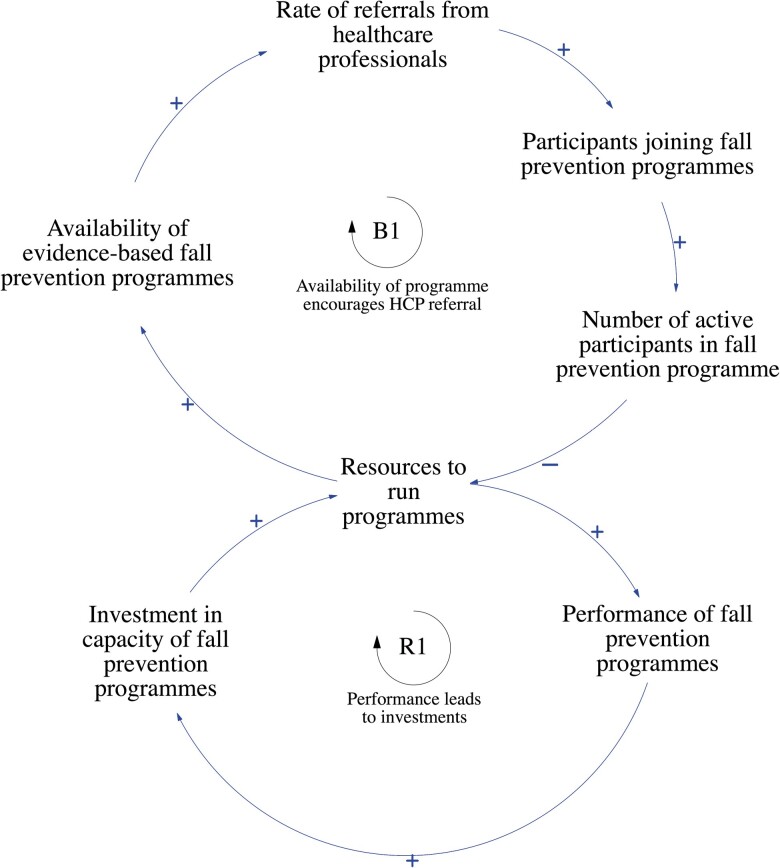
Health care provider factors influencing clinicians’ referrals. B1: Balancing Loop 1, R1: Reinforcing Loop 1.

Two loops illustrated how health care provider factors influenced the rate of referrals from HCPs (Figure 2).

Stakeholders identified that the main driver of referral rates made by HCPs is the availability of evidence-based fall prevention programs. However, with more participants in programs and limited resources available, this would adversely affect the availability of programs. This negative feedback is described in the balancing loop, B1. The stakeholders reported on the factors affecting the availability of evidence-based fall prevention programs. They included: (1) the availability of tailored components, (2) infrastructure, and (3) manpower. *Stakeholders mentioned that one-stop tailored programs were not widely available in community-based fall prevention programs in Singapore. A physiotherapist stated that a diverse range of components should be available at different program centers for customizations to be made based on the patient’s condition. A geriatrician also noted that there is a lack of integrated programs that begin interventions from home, that progress into center-based programs as patients improve.* Moreover, all stakeholders agreed that necessary infrastructure such as equipment and facilities, and trained manpower should be available to increase the availability of evidence-based programs as well.

Other identified factors affecting the rate of referrals from HCPs were (1) clinicians’ perception toward fall prevention programs and (2) clinicians’ awareness of programs they can refer to. *Stakeholders mentioned that not all doctors prioritize fall prevention in the clinic due to competing demands and time constraints. As such, many patients may not be referred to fall prevention programs should doctors choose to focus on other competing health needs.* Furthermore, it was reported by geriatricians that fall prevention is often not widely practiced in other specialties beyond geriatric medicine. Several doctors shared that available programs are poorly referred by clinicians, or that doctors are often unaware of the programs they can refer patients to besides physiotherapists.

The reinforcing loop, R1, describes how the performance of fall prevention programs leads to greater investments and resources for fall prevention programs, which can eventually increase performance. The performance of fall prevention programs is defined as the overall experience of the program, which includes program efficacy and patient satisfaction. Stakeholders reported that investments in fall prevention capacity are driven by (1) funding from national agencies and (2) copayment by individuals. Funding from national agencies is determined by meeting corporate key performance indicators (KPIs). *Stakeholders shared that due to the way KPIs are structured, physiotherapists may have to discharge patients for more patients to enter programs.* Competing priorities among providers (KPI-driven vs person-centered care) and current KPIs may negatively reflect the performance of programs. In addition, copayment by individuals is determined by patients’ willingness to pay for programs. This is influenced by patient factors, such as (1) patients’ value perception of fall prevention programs and (2) the cost of programs.

### Patient Factors Influencing Participant Engagement in Fall Prevention Programs

**Figure 3.**
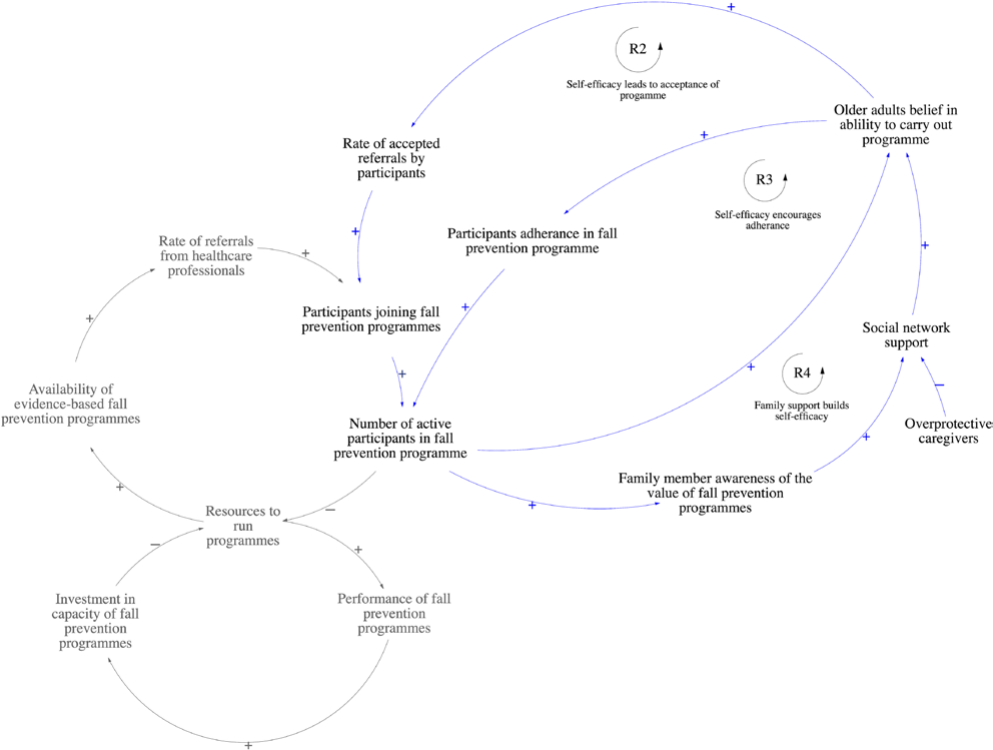
Older adults’ self-efficacy in carrying out programs implicating joining and adhering fall prevention programs. R2: Reinforcing Loop 2, R3: Reinforcing Loop 3, R4: Reinforcing Loop 4.

According to stakeholders, three feedback loops defined ways self-efficacy can lead to accepting referrals and adhering to fall prevention programs (Figure 3). Older adults’ self-efficacy for programs determines their response toward doctors’ referrals to fall prevention programs (R2), as well as their adherence to the program (R3). Stakeholders also identified that family support is one of the key drivers of older adults’ self-efficacy (R4).

According to stakeholders, participants’ self-efficacy for programs is influenced by: (1) their intrinsic motivation for exercise, (2) exercise self-efficacy, and (3) fear of falling. *Stakeholders shared that patients who readily join programs and adhere to them typically prioritize exercise and embrace the idea of healthy aging. On the other hand, those who are averse to joining programs have low exercise self-efficacy and are afraid of falling.* In addition, informed family members can encourage, supervise, and support older adults’ enrollment into programs. This increased social support can strengthen older adults’ belief in their abilities to carry out programs, promoting the rate of accepted referrals and long-term adherence. However, overprotective caregivers may restrict older adults from joining programs for fear of a future fall.

**Figure 4.**
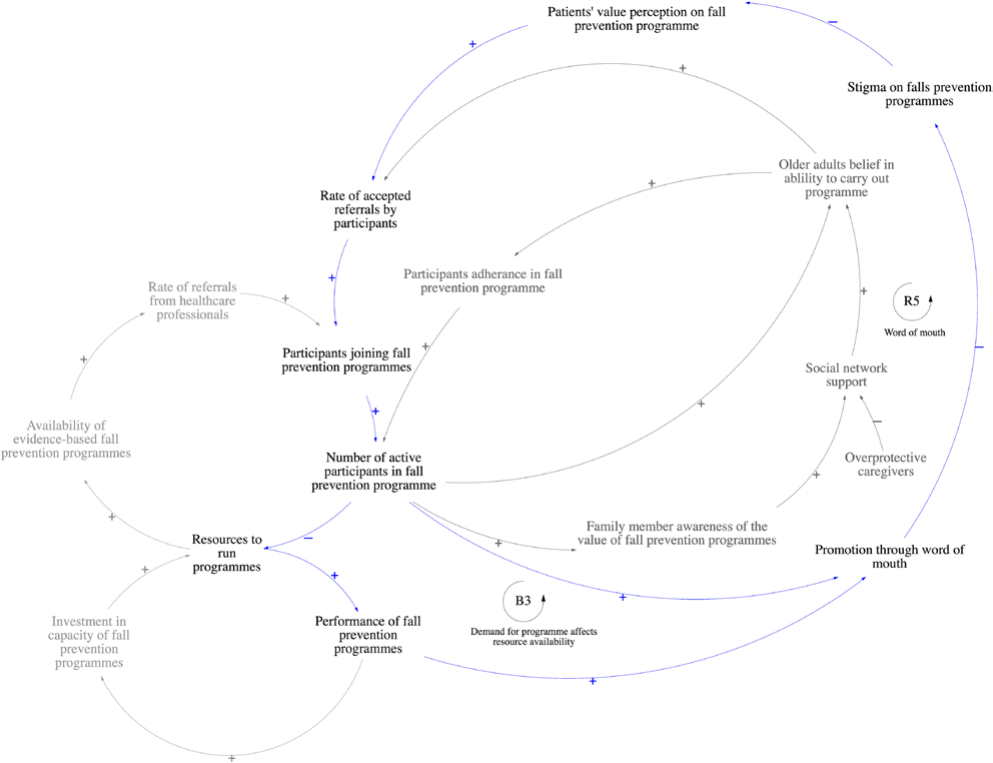
Promotion of fall prevention programs through word of mouth influences the rate of referrals accepted by participants. B3: Balancing Loop 3, R5: Reinforcing Loop 5.

Promotion through word of mouth affects the rate of older adults accepting referrals into programs (Figure 4). *Stakeholders shared that being labeled as a faller is stigmatized among older adults in the community. They observed that older adults actively avoid being labeled as a faller as it is perceived to be associated with old age.* As such, with more participants in fall prevention programs, and promoting the program through word of mouth, this may reduce the stigma on fall prevention programs and change the value perception of fall prevention programs, leading to more older adults accepting referrals to join programs (R5). The promotion of the program through word of mouth is also directly affected by the overall performance of the program. A lack of resources to run programs due to oversubscription may adversely affect program performance. This may eventually result in fewer participants accepting referrals (B3).

Stakeholders shared that patients’ value perceptions are influenced by fatalistic beliefs about aging and knowledge about fall prevention programs. Physiotherapists and occupational therapists shared that *falling is perceived as part of aging and that it cannot be prevented, hence older adults see no real value in participating in fall prevention programs.* Additionally, patient knowledge is affected by their awareness of their own fall risk, and awareness of the severity and consequences of a fall. *Stakeholders hypothesize that health literacy plays an important role in shaping patient attitudes, as they observed that patients who are typically more educated have a better understanding of fall prevention.*

**Figure 5.**
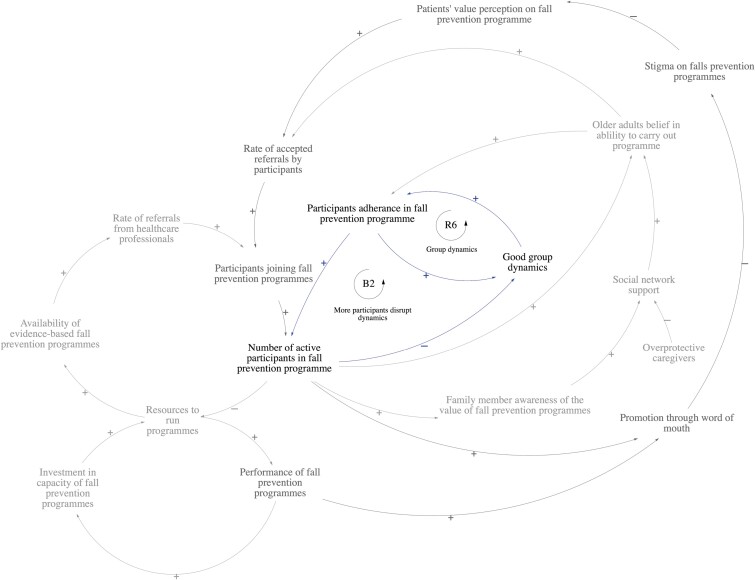
Good group dynamics promote adherence to fall prevention programs. B2: Balancing Loop 2, R6: Reinforcing Loop 6.

From their experiences running fall prevention programs, stakeholders emphasized the importance of good group dynamics in directly influencing adherence to programs (Figure 5). Good group dynamics would promote adherence to programs (R6). However, with more participants joining programs, this may adversely affect group dynamics due to groups being formed with participants with clashing personalities, preferences, and languages (B2).

Group dynamics are influenced by (1) peer involvement, (2) community-centric class setup, and (3) language barriers. Peer involvement in the form of peer-led facilitators can help build group cohesion. Having peer involvement inculcates a strong sense of ownership, which can promote adherence to programs. A community-centric class design also promotes participant adherence. *Two stakeholders shared that in a previous fall prevention study, there was a space allocated for participants to interact before and after the program. They mentioned that such a design not only encourages good group dynamics but also facilitated the formation of social networks.* Furthermore, language barriers impede group dynamics due to the lack of communication and operational challenges. *Stakeholders shared that in group settings, oftentimes, participants who communicate in different languages would exercise separately from the main group with a help of a translator by the side.*

**Figure 6.**
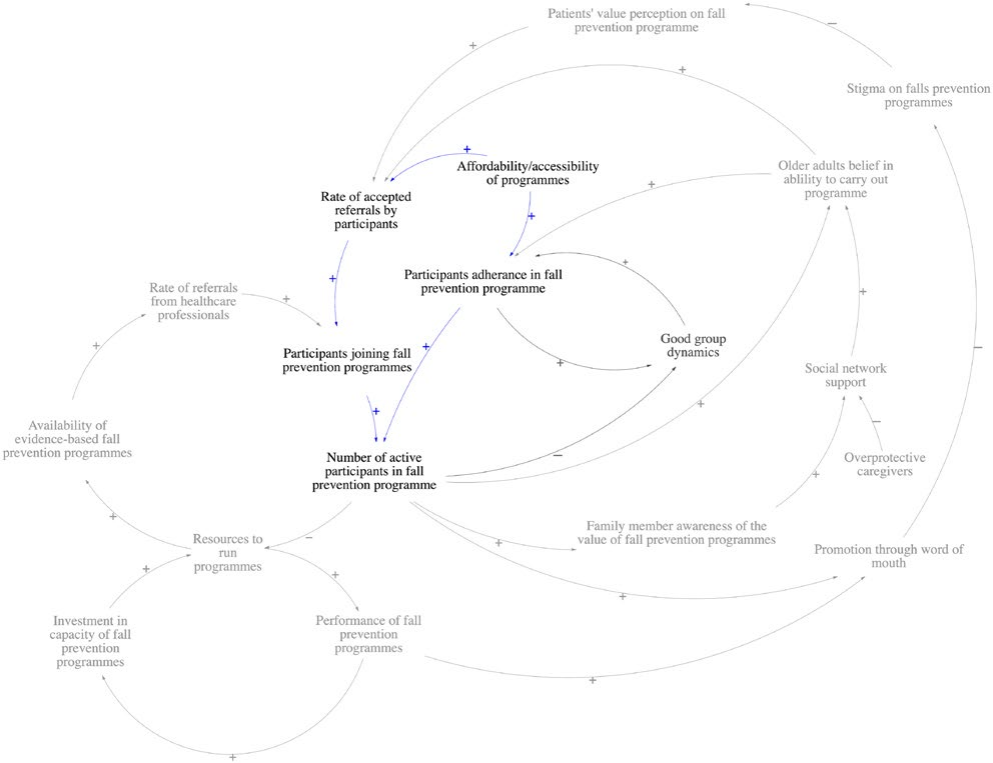
Affordability and accessibility influencing both rates of accepted referrals and long-term adherence to fall prevention programs.

Lastly, the affordability and accessibility of programs influence both participants joining fall prevention programs and adhering to programs (Figure 6). Stakeholders mentioned that there is a barrier to entry for certain types of programs due to cost. *For example, home-based programs are usually more expensive, hence dissuading potential participants who were willing to join programs but would prefer to do so in the comfort of their homes due to mobility issues or personal preferences. Furthermore, a geriatrician mentioned that household means-testing can sometimes make access to center-based exercise programs difficult as well.* Accessibility of programs is influenced by: (1) the availability of accompanying caregivers, (2) distance to the program center, (3) inappropriate urban outdoor environment, and (4) COVID-19 disruptions. According to the stakeholder group, older adults tend to be reliant on their caregivers for decision making, financial support, and mobility. *However, stakeholders noted that not all older adults require an accompanying caregiver. The younger older adults (i.e., 60–74 years old), tend to be more educated, can travel to program centers, and make decisions independently.* Stakeholders also mentioned that distance is a huge factor in influencing the accessibility of programs. The closer the program center is to the older adults’ place of residence, the more likely they are to participate. Moreover, the urban outdoor environment is also another barrier to accessing programs. The current outdoor environment is not mobility-aid friendly, discouraging older adults from leaving their homes. Finally, most recently, disruptions due to COVID-19 public health measures are a barrier to participation. *Stakeholders explained that after avoiding group activities for almost 2 years, some older adults are unwilling to participate in group exercise activities again.*

## Discussion

### Key Leverage Points and Archetypes Identified

The dynamic relationship between facilitators and barriers in implementing fall prevention programs was reflected in the CLD above. Based on the insights generated, key leverage points for interventions have been outlined to guide future implementation strategies.

The GMB exercise generated three major insights. Firstly, the performance of fall prevention programs can promote greater investments and resources to run programs. This can result in the greater availability of one-stop comprehensive person-centered programs, and more referrals from HCPs. However, should more participants join and engage in programs, this may result in overcapacity and affect the performance of programs. This insight is referred to as the “Limits to Growth” archetype (Braun, 2002; Figure 2), where efforts to invest in fall prevention programs may be successful in increasing performance in the initial stages, but an overcapacity eventually disrupts the growth.

Next, more participation in programs can gradually shift mindsets as the narrative of fall prevention programs can change through word of mouth. However, an overcapacity of participants may affect the performance of programs, which would adversely affect promotion through word of mouth. Similarly, these insights lead to the identification of the “Limits to Growth” archetype (Braun, 2002), demonstrated by the competing loops implicating the rate of accepted referrals by participants (Supplementary Figure 5).

Good group dynamics can promote adherence to fall prevention programs. However, with more participants in programs, this may unintentionally disrupt group dynamics due to issues like group size, clashing personalities, and language differences. These insights lead to the identification of the “Fixes the Fail” archetype (Braun, 2002), where group dynamics can promote adherence to programs but may be unintentionally disrupted due to the increasing number of active participants (Supplementary Figure 6).

### Policy Recommendations

**Figure 7.**
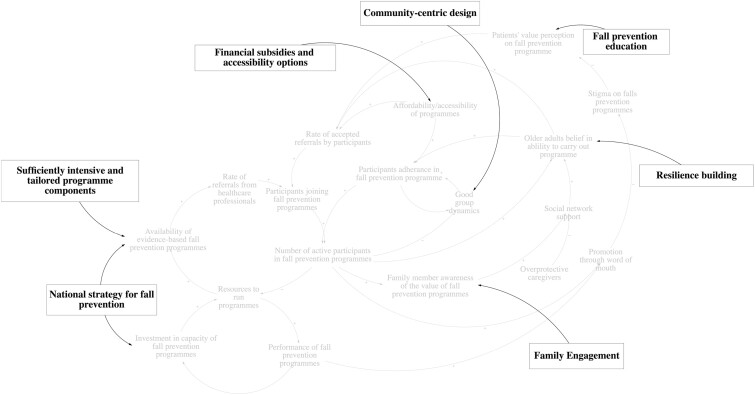
Suggested recommendations from stakeholder discussion targeting key leverage points.

The policy recommendations serve to increase the promoting (virtuous) loops and break the inhibiting (vicious) loops. Suggested recommendations targeting specific facilitators and barriers are summarized in Figure 7 and Table 2.

**Table 2.** Leverage Points for Intervention

Domains	Leverage point for intervention	Potential interventions
Program characteristics	Availability of evidence-based fall prevention programs	National strategy for fall prevention Capacity building for greater evidence-based fall prevention programs Systematic screening process followed by sufficiently structured, intensive, and customizable programs in both individual and group settings Structured and consistent referral pathways Guided by the national falls playbook
	Group dynamics	Socially conducive design Enabling environment Goal setting
Resource allocation and capacity planning	Corporate key performance indicators (KPIs)	Key stakeholders from diverse backgrounds need to restructure current KPIs Targets should be realistic and patient-centered
Patient factors	Patient attitude and knowledge	Fall education for participants and wider public Nationwide fall education campaign Selective messaging approaches to engage different groups in the community
	Older adults’ self-efficacy to carry out programs	Educate participants on value and specific purpose of activities Resilience-building strategies Family engagement and outreach
	Family member awareness of the value of prevention programs	Family engagement and outreach
Affordability and accessibility of programs	Affordability	Financial subsidies to be made available and accessible for participants
	Accessibility	Transportation services Facilitators and therapists to be readily available to assist

A key insight attained was that older adult fallers are largely considered a homogenous population in current community-based intervention programs in Singapore. In reality, they are a heterogeneous group of individuals, with various risk factors characterizing different profiles of fallers (Pereira & Kanashiro, 2022; Xu et al., 2022). An older adult with falls typically has complex health needs and multiple underlying disorders like sarcopenia, osteoporosis, cognitive impairment, cardiovascular syncope, etc. (Duque & Kiel, 2016). There is generally a lack of high-profile societal importance on fall prevention compared to these other pathologically defined health needs, as a result, fall prevention often must compete with these priorities. In addition, identifying the risk factors and optimal approach to address them continues to be a complex process, especially with the continually updating literature on fall risk factors. Moreover, decisions to join fall prevention programs are influenced by factors such as personal perceptions, social support, convenience, and cost (Fernandes et al., 2021; Vincenzo et al., 2022). Therefore, fall prevention must be understood as a cross-disciplinary issue that is not inevitable with aging. In addition, discussions should include increasing human resources, capacity, and optimal funding mechanisms for HCPs involved in fall prevention programs. This GMB provided insight that current fall prevention programs were carried out in silos. Hence, there is a strong need to integrate efforts and shift toward a higher level of coordination between multidisciplinary teams and complementary provider groups moving forward.

### National Strategy for Fall Prevention

There is a strong urgency to develop an overall national strategy for fall prevention. From discussions, there is a general agreement about the limited national agenda on falls. Establishing a falls workgroup would be the first step to organizing efforts, consolidating current learnings across all health care clusters in Singapore and driving the agenda of national falls prevention. The workgroup should integrate existing frameworks and establish appropriate frameworks to guide risk screening, referrals, prevention, and management programs.

The workgroup needs to consist of a multidisciplinarian team of professionals, comprising clinicians from various specialties (e.g., rehabilitation therapists, nurses, pharmacists, cardiologists, neurologists, dieticians, endocrinologists, and geriatricians), researchers, and policymakers. It should be chaired by individuals from different organizations to ensure consistent leadership and to maintain diversity and continuity of work in the long run. The focus of the workgroup would be to harmonize the understanding of falls across all stakeholders and recommend concrete clinical and community guidelines on the various facets of fall prevention. A comprehensive evaluation of fall prevention programs should first be conducted to understand the gaps between research and translation. The GMB highlighted that there is overwhelming evidence from research studies and geriatric clinics on the approaches to fall prevention in Singapore; demonstrating how Singapore is in a good position to translate findings in the community by building on existing capacities. However, several gaps remain, such as the feasibility of implementation on a health-cluster level, maintenance of programs, and the overall cost-effectiveness of fall prevention programs. A national falls playbook with specific guidelines should be developed after evaluation. The playbook serves to inform decision-makers when adapting fall prevention programs at their respective institutions. At the same time, it should incorporate flexibility, to enable decision-makers to shape programs according to their targets, resources, and capacity. Stakeholders also maintain that a mixture of top-down and bottom-up approaches should be adopted. However, the formation of a workgroup and establishing necessary agendas and guidelines should be done before introducing government involvement.

Establishing a national fall prevention strategy would pave the way for the implementation of various fall prevention programs. A fall prevention program should be efficacious, feasible for implementation, and cost-effective. An organized national fall prevention strategy would work to increase resources available for programs and promote the availability of evidence-based fall prevention programs. Furthermore, the national strategy should also restructure current KPIs, to ensure that targets are realistic and patient-centered and that resources are utilized appropriately. The availability of evidence-based fall prevention programs should also include a structured screening process. Stakeholders shared that currently there is no systematic screening process for fall risk. As such, the national strategy should include identifying a valid, reliable, quick, and easy-to-administer screening tool to be used in the community. Existing clinical practice guidelines (CPG; HPB-MOH Clinical Practice Guidelines (1/2015), 2015) on fall prevention should also be reviewed on a more regular basis, with greater specificity on the criteria or tools for fall risk assessments. Recommended tools can be integrated with existing screening frameworks, such as the Integrated Care for Older People tool that screens for frailty. At the same time, a uniform criterion for patient referral should be developed alongside fall risk assessment guidelines. CPGs can detail specific programs for clinicians to refer patients to. This ensures that clinicians have a structured and consistent referral pathway to follow through after risk assessment. This is especially important for a multidisciplinary care team. Referral pathways could also be more inclusive to include patients with various conditions such as sensory difficulties and cognitive impairment, as these patients are at higher risk for falls. Further discussions need to take place to ensure fall prevention programs are available for these individuals in strong consideration of their current abilities, safety, and possible progression. Stakeholders also shared that current health care clusters require guidance to administer systematic screening assessments, hence it is also necessary to develop guidelines and additional training for relevant personnel in the health care clusters. Lastly, fall prevention programs should also be accompanied by consistent program evaluations in both short and long term, to ensure successful implementation in the long run. Some areas for evaluation include the effectiveness of programs, financial modeling of programs, and participant satisfaction.

### Fall Prevention Programs

#### Program components

Insights from stakeholders indicated that current programs are insufficiently intensive and are not targeted enough to create an experience of value. For programs to be efficacious, they need to have a degree of customization. Although this may be challenging to accommodate in a group setting, it would be beneficial to create programs specially designed to incorporate principles of progression, for those who improve quickly and require more intensive exercises, as well as principles of regression, for participants who are unable to manage such high intensities. Balancing these principles would be pivotal in ensuring adherence to programs in the long term.

#### Socially conducive environment

Design features in community-based fall prevention programs must be deliberate in promoting interaction between participants. This provides an arena for older adults to build their social networks, learn from others’ experiences, and progress together. At the same time, fall prevention programs should be an enabling environment, where participants are comfortable with taking ownership of their health. Facilitators can begin incorporating this by working together with older adults to set realistic goals for improvements (Mahoney et al., 2017). A socially conducive environment can promote long-term adherence to programs.

#### Building self-efficacy

Furthermore, the CLD suggests the importance of capitalizing on the reinforcing nature of older adults’ self-efficacy, and how engaging family members and caregivers would be beneficial in promoting self-efficacy among older adults. Trained facilitators (Jones et al., 2016; Mahoney et al., 2017; Shubert et al., 2014) in fall prevention programs need to educate participants on the value and specific purpose of their activities. Furthermore, fall prevention programs can also incorporate resilience-building (Faes et al., 2010; Matchar et al., 2017; Tchalla et al., 2014) to impart various strategies to build resilience—specifically to encourage participants to adhere to fall prevention programs should they find it a daunting task. Facilitators or outreach staff should actively engage family members and caregivers and educate them about the dangers of being overprotective (Matchar et al., 2017; Xu et al., 2019).

#### Fall education

The CLD also identified patients’ value perception as an important leverage point for intervention. Falls education would be important in raising awareness of their own fall risk, the severity of falls, and the importance of fall prevention. Engaging older adults’ families and educating them on falls is an important aspect as well. In addition, community guidelines should be established to ensure the public is aware of the importance of fall prevention, and how to conduct simple risk assessments to understand their fall risk. Messaging practices such as positive versus negative messages or self- vs family-centric approaches would be effective in such a context and should be tailored to the profile of the recipients (Mikels et al., 2016) when communicating with specific groups in public.

#### Affordability and accessibility

Finally, fall prevention programs need to consider participants’ reliance on caregivers and provide options should caregivers be unavailable. Such examples include providing transportation services and having therapists/assistants to reassure participants and their caregivers that the program would be safe and beneficial. Working with providers such as older adult welfare voluntary organizations would be crucial in making these services available. Financial subsidies should also be available and accessible, whereas ensuring programs remain cost-effective for sustained implementation (Carande-Kulis et al., 2015; Matchar et al., 2019).

### Strengths and Limitations

Having a group of clinicians, allied health professionals and researchers, who play an active role in fall prevention, examine and discuss implementation challenges is a strength of this study. The active participatory approach through the GMB was key in encouraging in-depth discussion and cocreation of policy targets through shared consensus. It promoted strong urgency to advocate for the development of a comprehensive national strategy that encapsulates the various facets of fall prevention. Furthermore, by employing system dynamics methods, the relationships and interactions between factors can be elucidated and visualized in the conceptual model.

However, we acknowledge that there are limitations to this research. This discussion of fall prevention only involved HCPs; additionally, many of the feedback loops developed involved HCPs’ perceptions of patient experiences regarding fall prevention. Although HCPs play an important role in managing fall prevention with patients and have intimate knowledge of managing health with patients, they cannot replace vital patient voices in their experiences with fall prevention. Patient perspectives are lacking in this current study; however, this study is the first of a series of studies in engaging stakeholders to reflect on fall prevention in Singapore. Future works are underway to engage with older adults to understand their perspectives as well as to identify discordance between HCPs’ and patients’ attitudes. Other stakeholders such as social and community providers and policymakers will be approached in future studies to ensure that a representative and comprehensive understanding of fall prevention has been captured. Although we recognize that these results may be unique and limited to Singapore, the insights gathered may be relevant for other Asian settings or multiethnic settings with developed health care systems. To the authors’ knowledge, this study is the first done in an Asian context.

## Conclusion

Through the GMB, stakeholders were engaged in discussing the challenges of fall prevention among older adults. This led to the development of a conceptual model to elucidate issues surrounding the implementation of fall prevention programs. Key leverage points have been identified and recommendations have been put forward as well. The group acknowledged that there is a wealth of evidence in various geriatric clinics and research groups, but they have yet to be properly implemented on a national scale. Hence, it is imperative to consider developing a comprehensive national fall prevention strategy.

Moving forward, an independent set of stakeholders involving older adults, other HCPs, social and community providers, and policymakers will be engaged to verify the concept model for face and content validity. Furthermore, to generate more insights to support the development of a national fall prevention strategy, future work would involve developing a credible quantitative model for simulation. The CLD generated from this study would be further enhanced to generate the quantitative model. In conclusion, the model generated and future additional work would be useful to guide future approaches to fall prevention interventions.

## Supplementary Material

igad077_suppl_Supplementary_Materials
