# Supplementary material for: Reducing Falls Among Community-Dwelling Older Adults From Clinicians’ Perspectives: A Systems Modeling Approach
Source: Innov Aging. 2023 Jul 20;7(7):igad077. doi: 10.1093/geroni/igad077 (PMC10484166; doi:10.1093/geroni/igad077)
Supplement: igad077_suppl_Supplementary_Materials [file igad077_suppl_supplementary_materials.docx]

# Online Supplementary Material

**Figures and Illustrations**

Supplementary Figure 1. Key outcomes prioritised by stakeholders.

| Patient attitude | Patient knowledge | Patient perceptions |
| --- | --- | --- |
| *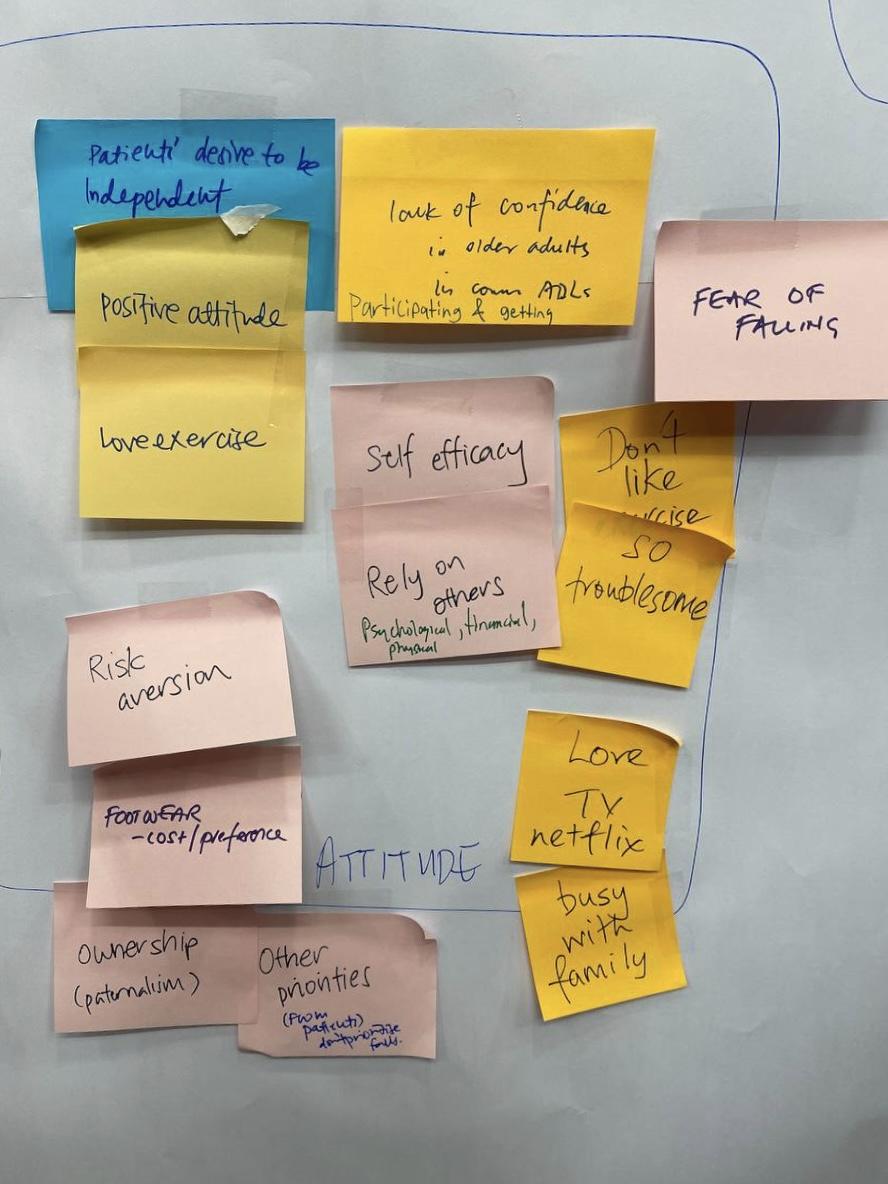* | *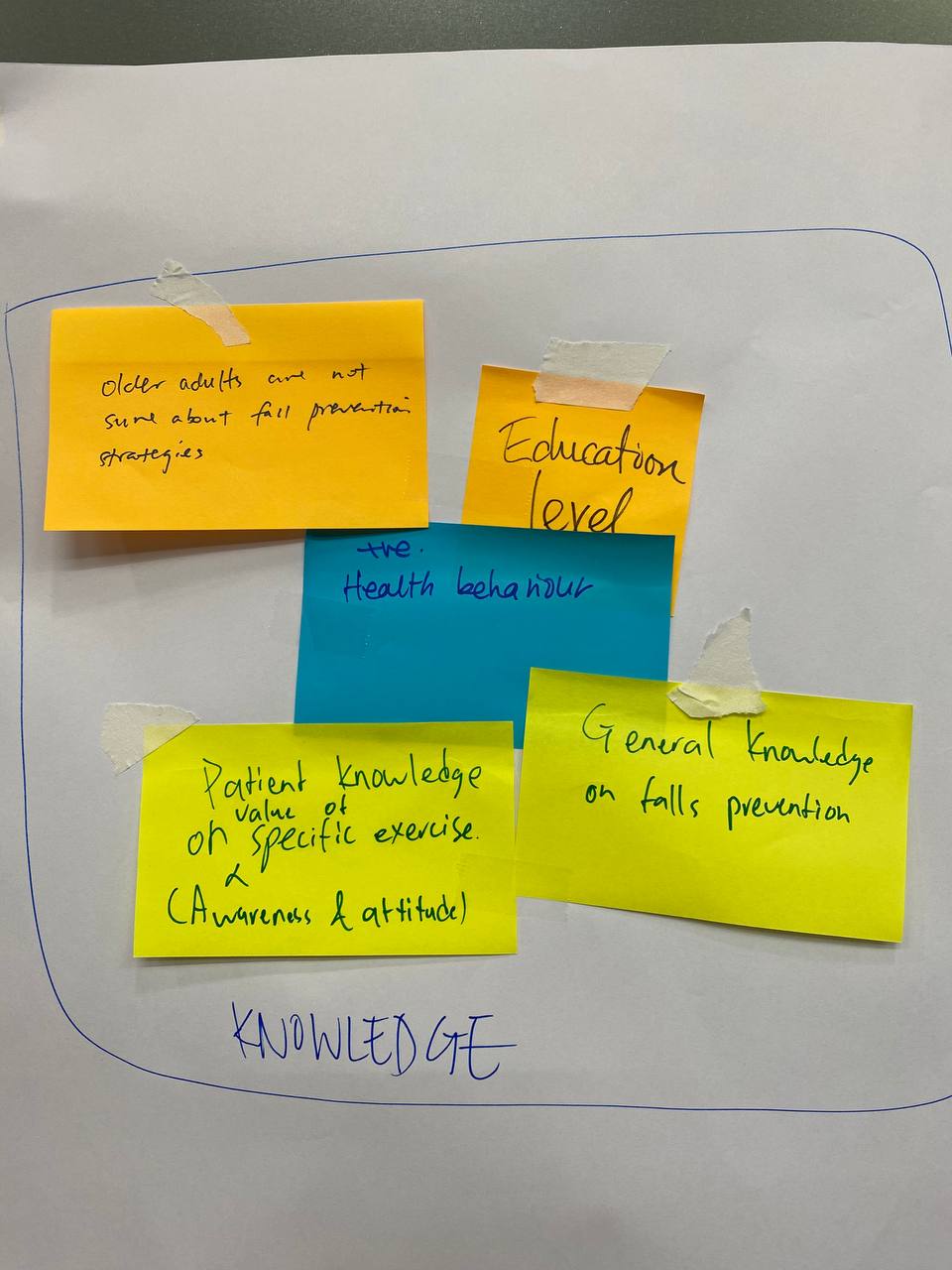* | *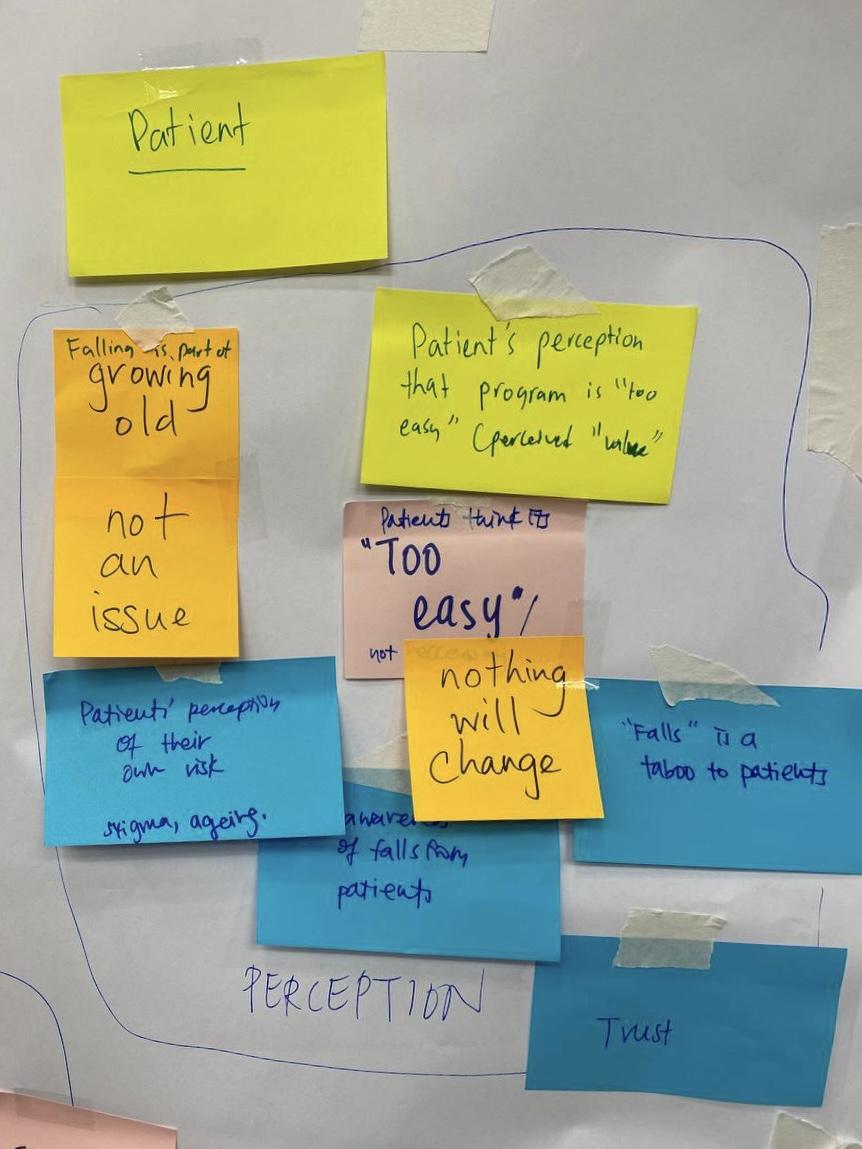* |
| Accessibility of programme | Availbility of programme | Characteristics of programme |
| *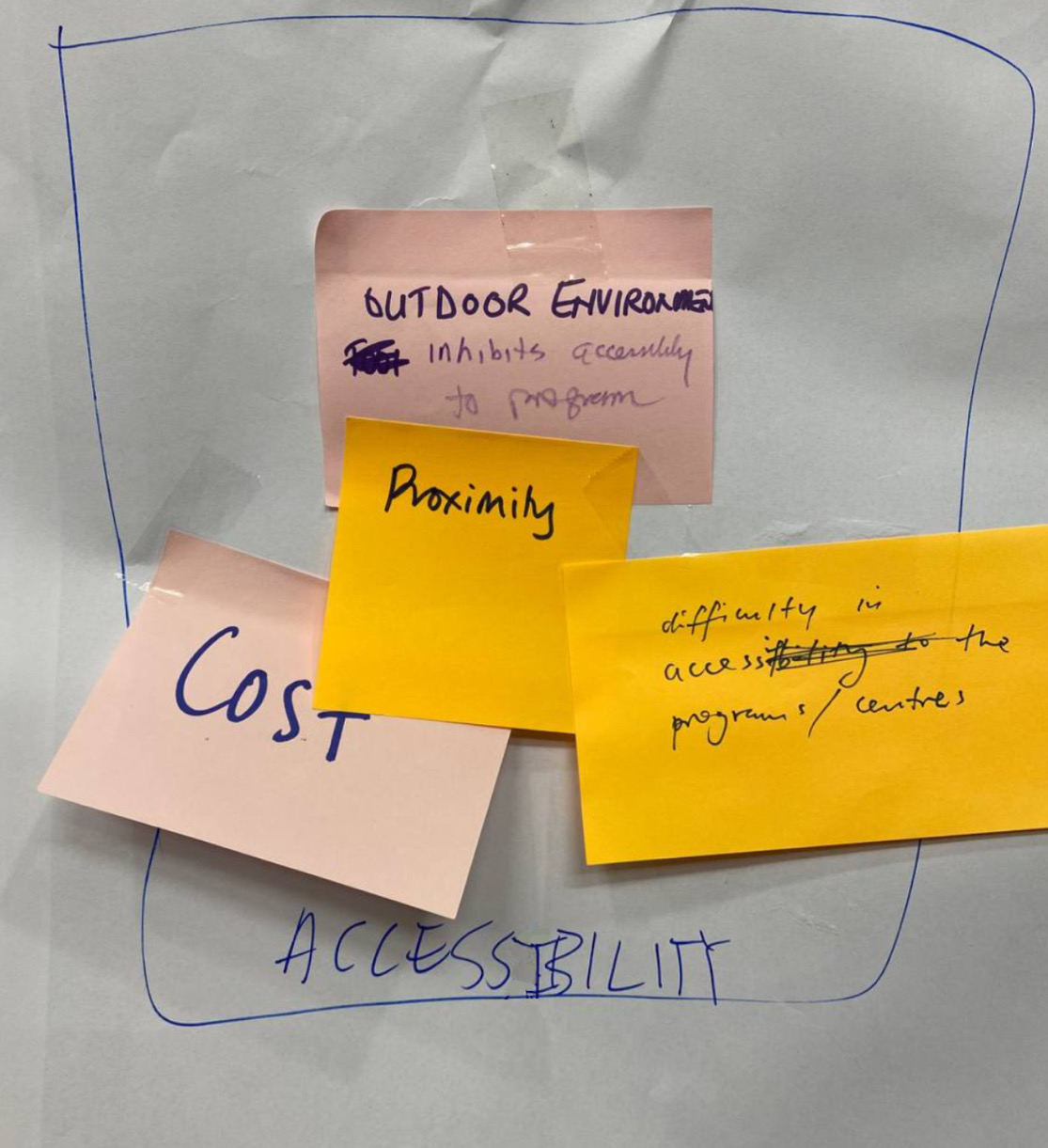* | *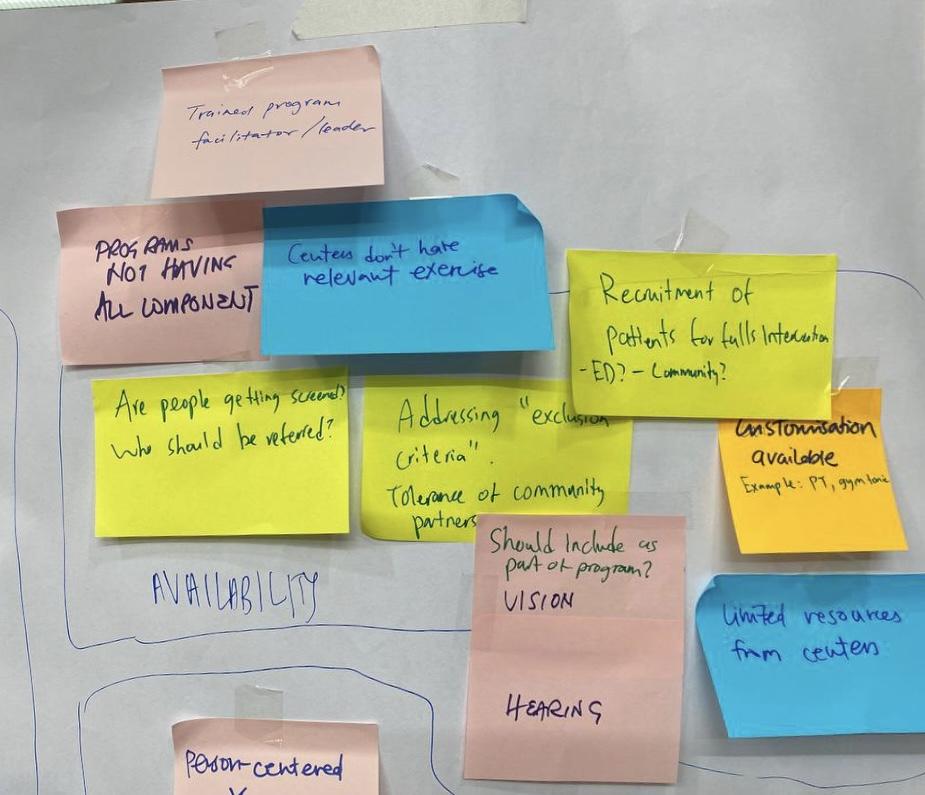* | *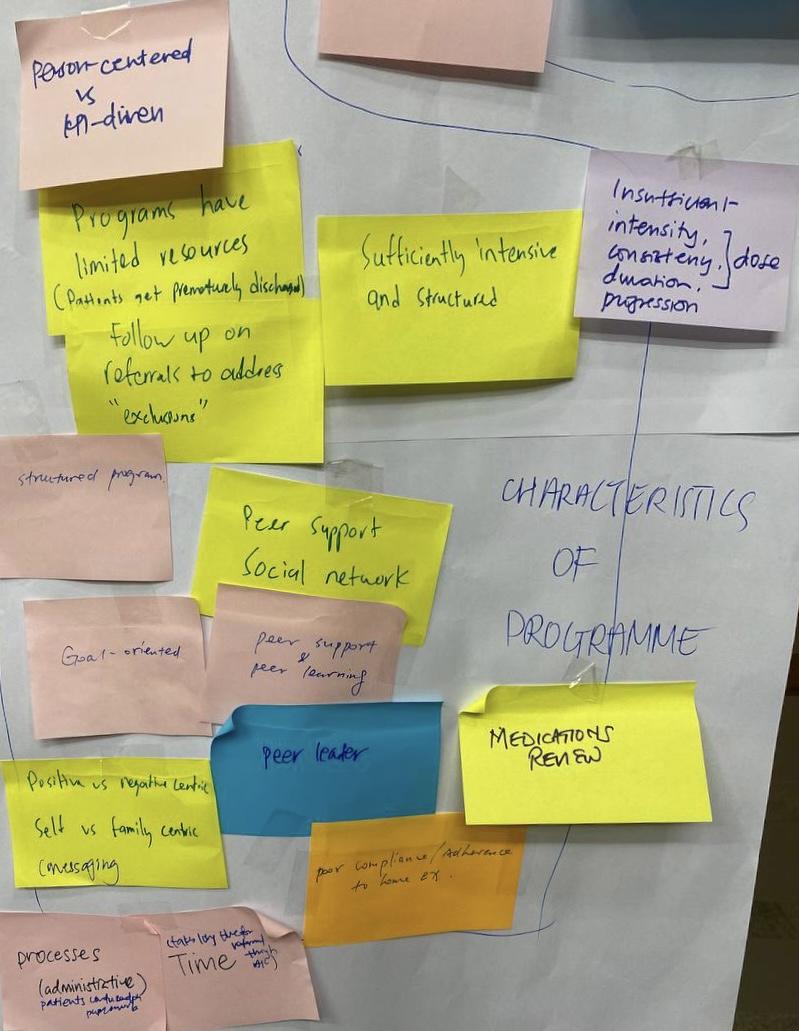* |
| Community-building | Family and societal norms |  |
| *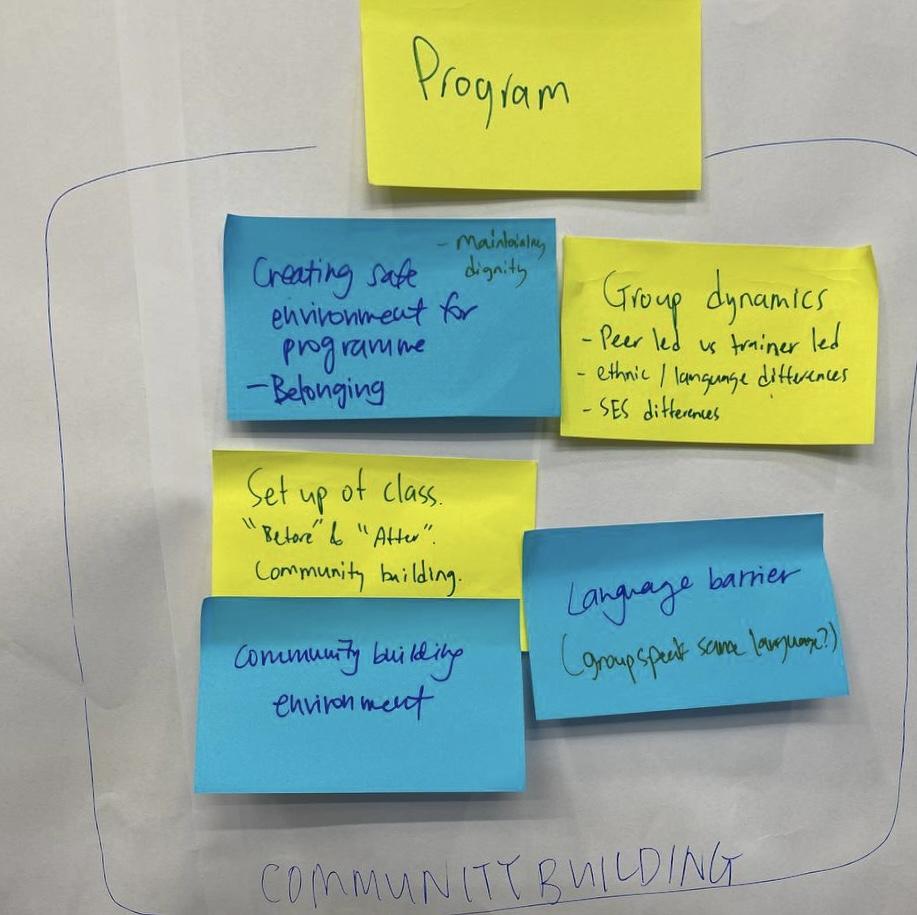* | *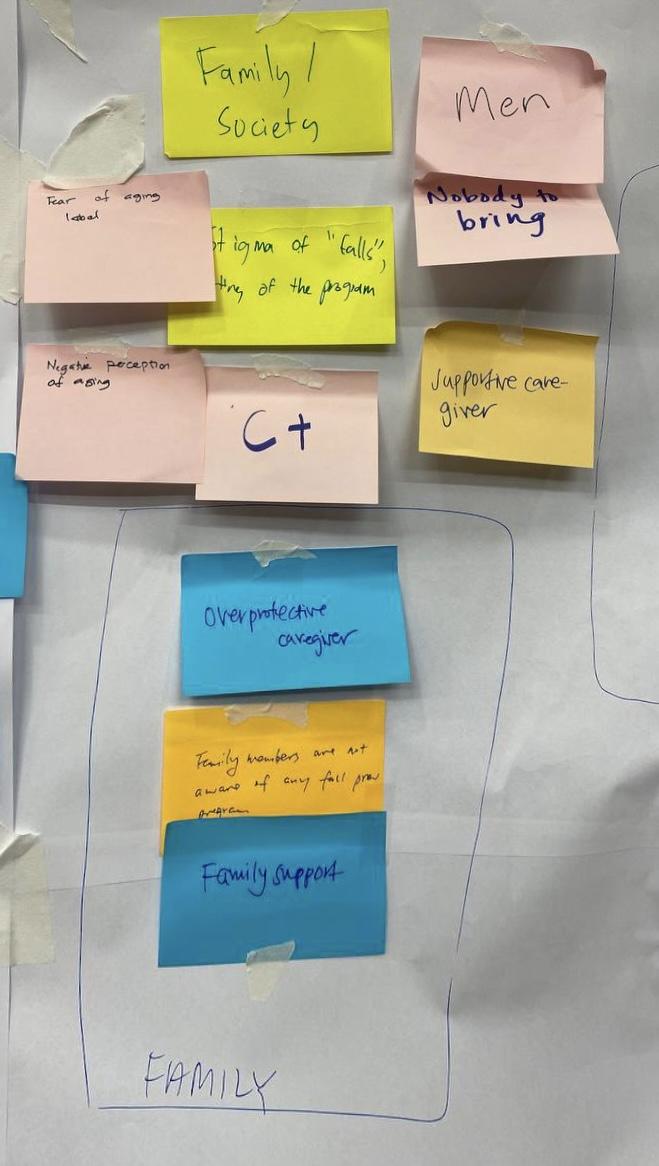* |  |

Supplementary Figure 2. Groups categorised after variable elicitation.

| *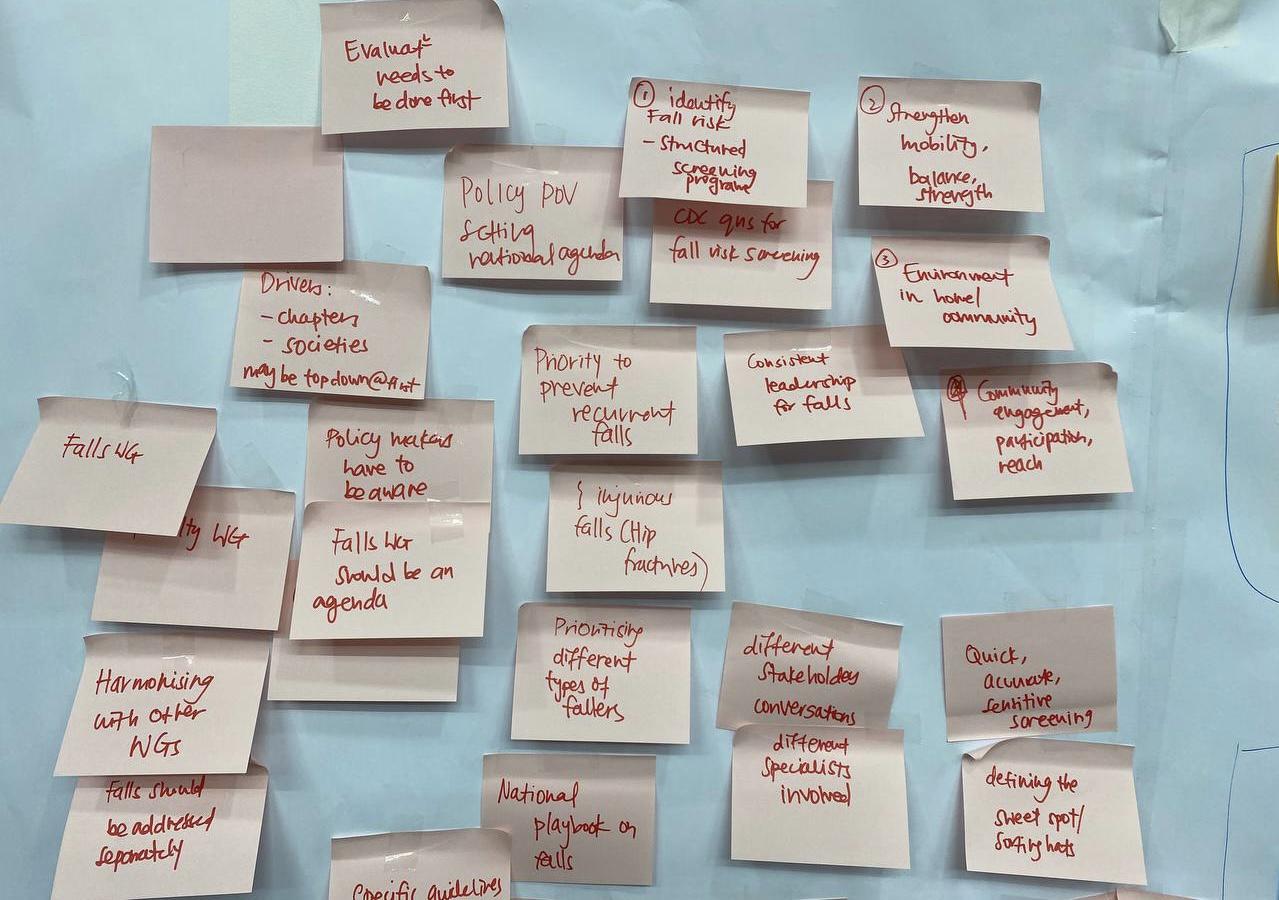* | *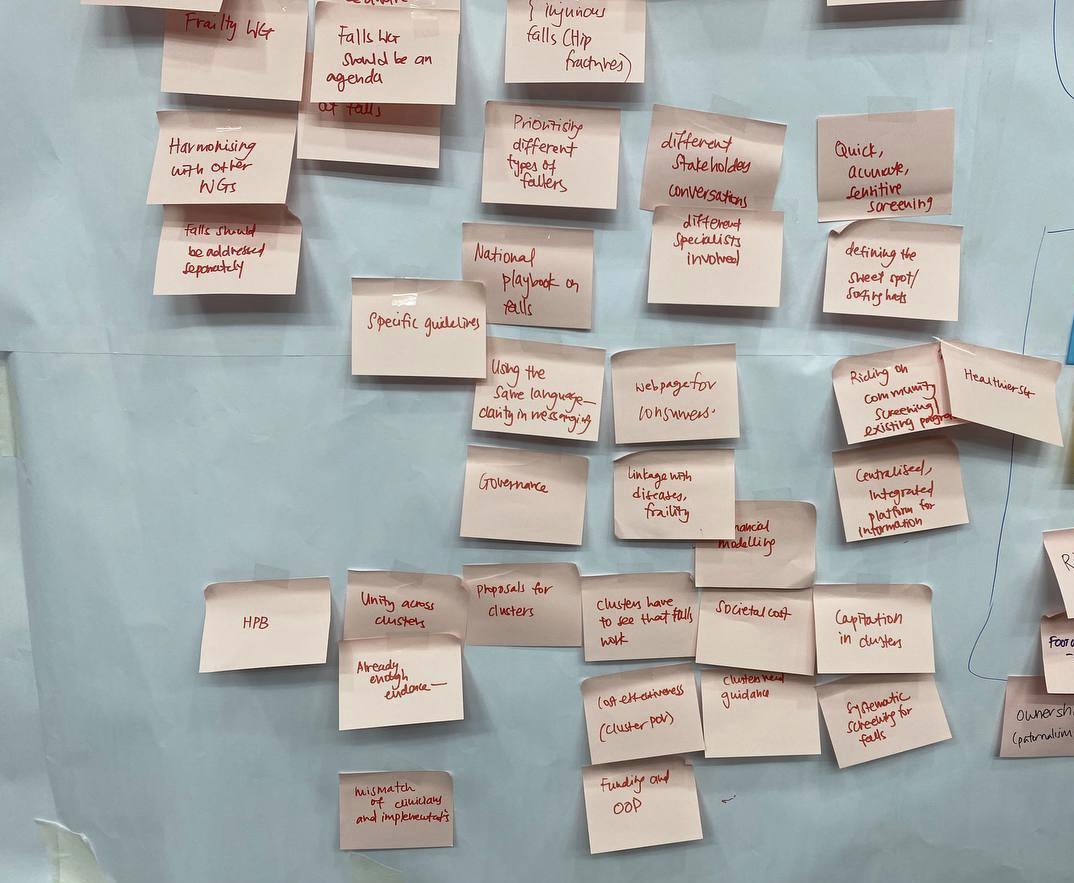* |
| --- | --- |

Supplementary Figure 3. Policy recommendations.

*
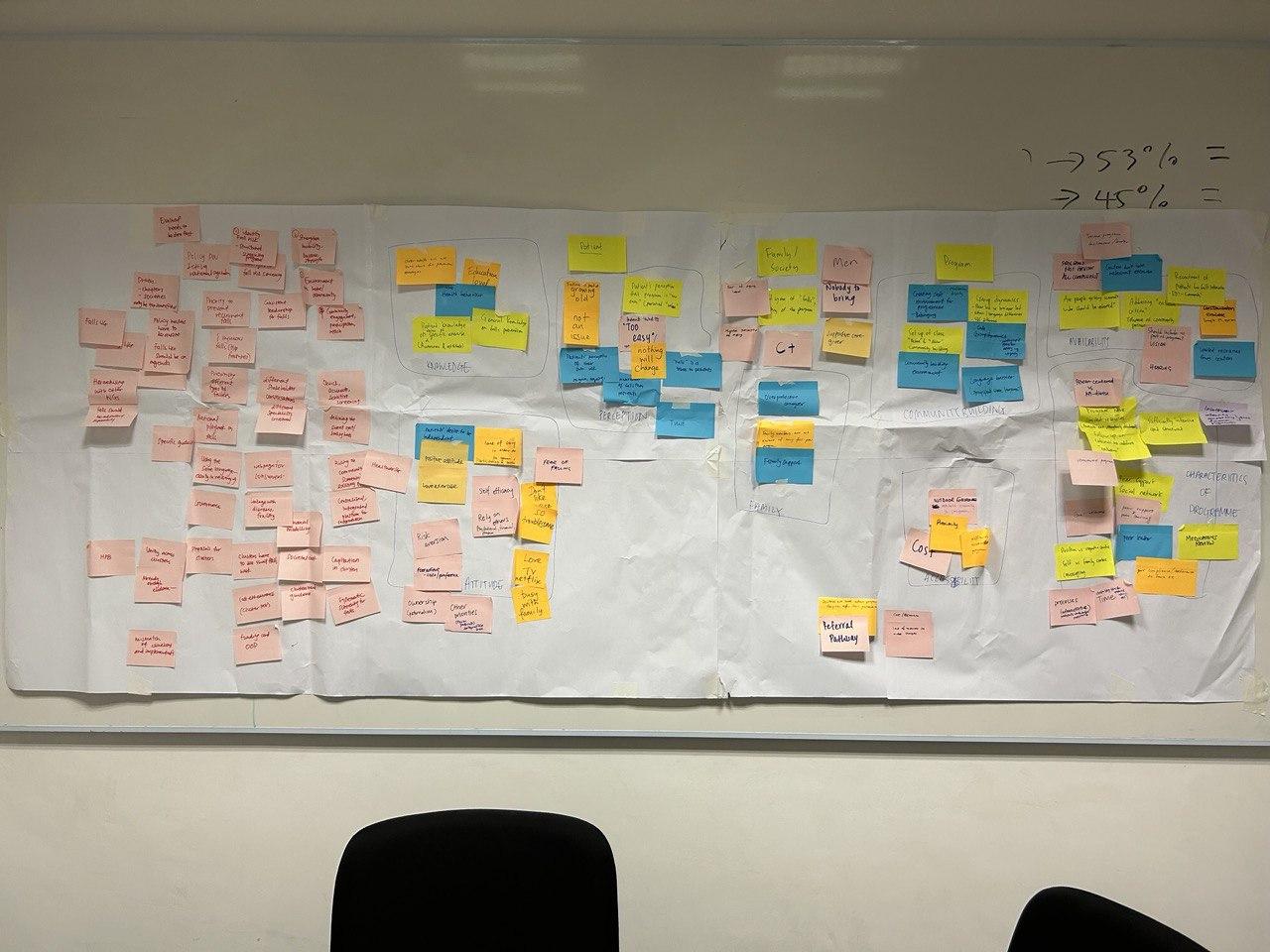
*

Supplementary Figure 4. Overview of variables for the concept model.

*
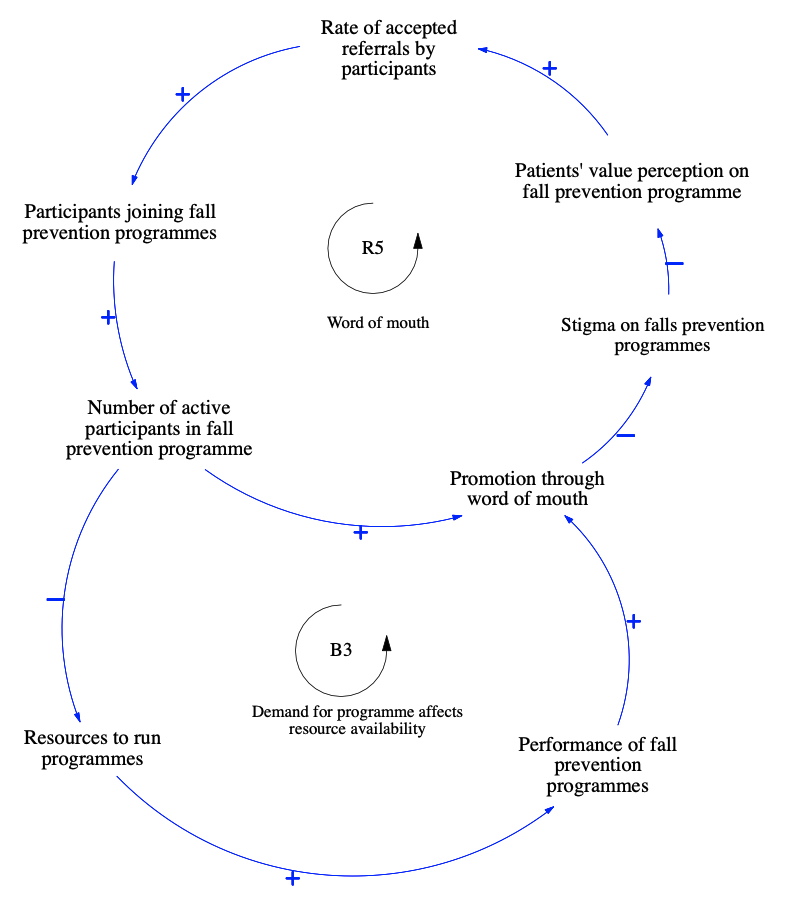
*

Supplementary Figure 5. Performance of programmes and word of mouth – Limits to Growth archetype adapted from Braun, 2002. R5: Reinforcing Loop 5, B3: Balancing Loop 3.

*
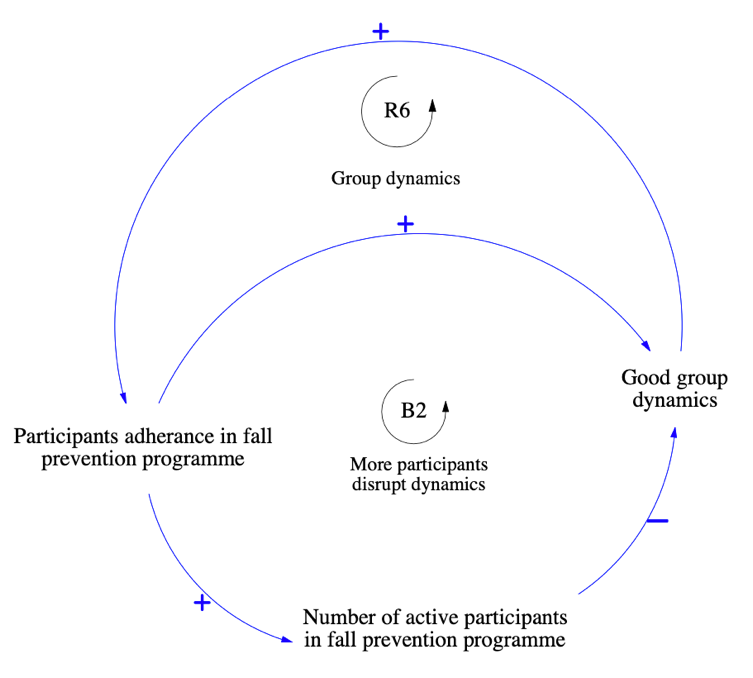
*

Supplementary Figure 6. Good group dynamics promote adherence to programmes, but more active participants may unintentionally disrupt group dynamics – Fixes the Fail archetype adapted by Braun 2002. R6: Reinforcing Loop 6, B2: Balancing Loop 2.

**Tables**

Supplementary Table 1. Sequence of activities for GMB.

| Agenda | Activity |
| --- | --- |
| Session 1 |  |
| Introduction and Overview | - Introduction of research team and stakeholders - Background and objectives of the GMB workshop |
| Persona Creation Icebreaker | - Introduction of the patient persona - Stakeholders were asked to share their tension points on fall prevention to create the clinician persona |
| GMB Exercise 1: Outcome Elicitation | - Stakeholders were asked to share key outcomes they were interested in regarding fall prevention and management - Vote to prioritise variables |
| GMB Exercise 2: Variable Elicitation | - - Elicit factors   - Coloured post-it notes were distributed for writing factors   - In a round-robin fashion, each stakeholder was asked to select a variable and describe why and how it is important   - Facilitators asked stakeholders to define variables and identify the causal relationship between factors   - Variables were clustered on the board and later categorised into groups |
| Session 2 |  |
| GMB Exercise 3: Exploring Policy Options | - Stakeholders were asked to explore policy recommendations to target facilitators and barriers identified - Stakeholders identified bottlenecks for possible interventions to improve the implementation of fall prevention programmes |
| Debriefing session and Close | - Insights from workshop were summarised - Shared next steps of the project |

Supplementary Table 2. Symbols in a Causal Loop Diagram.

| Symbol | Definition |
| --- | --- |
| *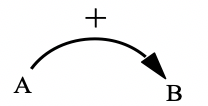* | A positive sign “+” indicates a positive relationship between variables A and B. In this case, an increase in variable A leads to an increase in variable B; or a decrease in variable A leads to a decrease in variable B. |
| *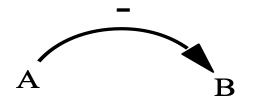* | A negative sign “-“ indicates an inverse relationship between variables A and B. In this case, an increase in variable A leads to a decrease in variable B; or a decrease in variable A leads to an increase in variable B. |
| *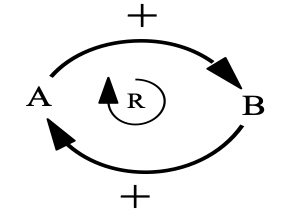* | “R” denotes that the feedback loop is a reinforcing loop. In a reinforcing loop, an increase in variable A leads to an increase in variable B, which leads to a further increase in variable A. Similarly, a decrease in variable A will also lead to a further decrease in variable A too. The reinforcing feedback loop results in exponential changes, where there may be exponential growth or decay. |
| *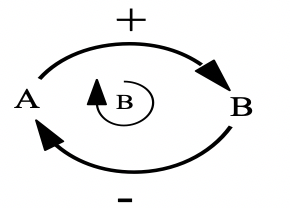* | “B” denotes that the feedback loop is a balancing loop. In this balancing loop an increase in variable A leads to an increase in variable B, which finally results in a decrease in variable A. Similarly, a decrease in variable A will also lead to a further decrease in variable A too. Over time, a balancing loop seeks to counteract changes to the system and the feedback loop will reach an equilibrium. |
